# Metabolic Analysis of Medicinal *Dendrobium officinale* and *Dendrobium huoshanense* during Different Growth Years

**DOI:** 10.1371/journal.pone.0146607

**Published:** 2016-01-11

**Authors:** Qing Jin, Chunyan Jiao, Shiwei Sun, Cheng Song, Yongping Cai, Yi Lin, Honghong Fan, Yanfang Zhu

**Affiliations:** Department of Life Sciences, Anhui Agricultural University, Hefei, 230036, China; CSIR- Indian Institute of Toxicology Research, INDIA

## Abstract

Metabolomics technology has enabled an important method for the identification and quality control of Traditional Chinese Medical materials. In this study, we isolated metabolites from cultivated *Dendrobium officinale* and *Dendrobium huoshanense* stems of different growth years in the methanol/water phase and identified them using gas chromatography coupled with mass spectrometry (GC-MS). First, a metabolomics technology platform for *Dendrobium* was constructed. The metabolites in the *Dendrobium* methanol/water phase were mainly sugars and glycosides, amino acids, organic acids, alcohols. *D*. *officinale* and *D*. *huoshanense* and their growth years were distinguished by cluster analysis in combination with multivariate statistical analysis, including principal component analysis (PCA) and orthogonal partial least squares discriminant analysis (OPLS-DA). Eleven metabolites that contributed significantly to this differentiation were subjected to t-tests (P<0.05) to identify biomarkers that discriminate between *D*. *officinale* and *D*. *huoshanense*, including sucrose, glucose, galactose, succinate, fructose, hexadecanoate, oleanitrile, myo-inositol, and glycerol. Metabolic profiling of the chemical compositions of *Dendrobium* species revealed that the polysaccharide content of *D*. *huoshanense* was higher than that of *D*. *officinale*, indicating that the *D*. *huoshanense* was of higher quality. Based on the accumulation of *Dendrobium* metabolites, the optimal harvest time for *Dendrobium* was in the third year. This initial metabolic profiling platform for *Dendrobium* provides an important foundation for the further study of secondary metabolites (pharmaceutical active ingredients) and metabolic pathways.

## Introduction

*Dendrobium* is a perennial herb in the family *Orchidacea* (*Dendrobium* Sw.) and is widely distributed in Australasia, Oceania and other tropical and subtropical areas [1,2]. In China, there are 74 *Dendrobium* species and two varieties [3], and nearly 50 of these species are used in medicine [4]. However, wild *Dendrobium* resources are threatened by extinction due to slow growth rates, habitat destruction and overexploitation. Thus, artificial, large-scale cultivation of medical *Dendrobium* has been developed. As valuable Chinese medicinal materials, *Dendrobium* species play important pharmacological roles with abundant polysaccharides, alkaloids, phenanthrenes, bibenzyls, and other biologically active substances [5,6]. However, the chemical constituents and contents differ significantly among different medicinal *Dendrobium* species. Some non-genuine *Dendrobium* is adulterated and many fake species referred to as “*Dendrobium*” are circulating in the market. This misrepresentation is not conducive to the safety and quality of medicinal *Dendrobium*, its clinical applications, or the healthy development of the industry. Therefore, an effective comprehensive method of *Dendrobium* germplasm identification and quality control is urgently needed.

*Dendrobium officinale* Kimura et Migo and *Dendrobium huoshanense* C. Z. Tang et S. J. Cheng are both commercially valuable, particularly *D*. *huoshanense* [7]. A comprehensive analysis of the chemical compositions of cultivated *D*. *officinale* and *D*. *huoshanense* and the differences in their metabolic components have not been reported. Metabolomics is the study of all low molecular weight metabolites within an organism or cell during a specific period of time by both qualitative and quantitative methods. Metabolomics has been widely used in the study of medicinal plants, including the identification of medicinal herbs [8], discrimination of origin [9], determination of harvest time [10], method of processing [11] and other factors. Metabolomic studies of *Dendrobium* metabolites have been limited.

In this study, a metabolic profile of *Dendrobium* was constructed using gas chromatography-mass spectrometry (GC-MS) combined with multivariate statistical analysis. The changes in the composition and content of metabolites, including sugars, alcohols, organic acids, amino acids and other metabolites, were studied in perennially cultivated *D*. *officinale* and *D*. *huoshanense* to identify biomarkers as a reference for the identification and quality control of *Dendrobium*.

## Materials and Methods

### Plant materials and reagents

**Fig 1:**
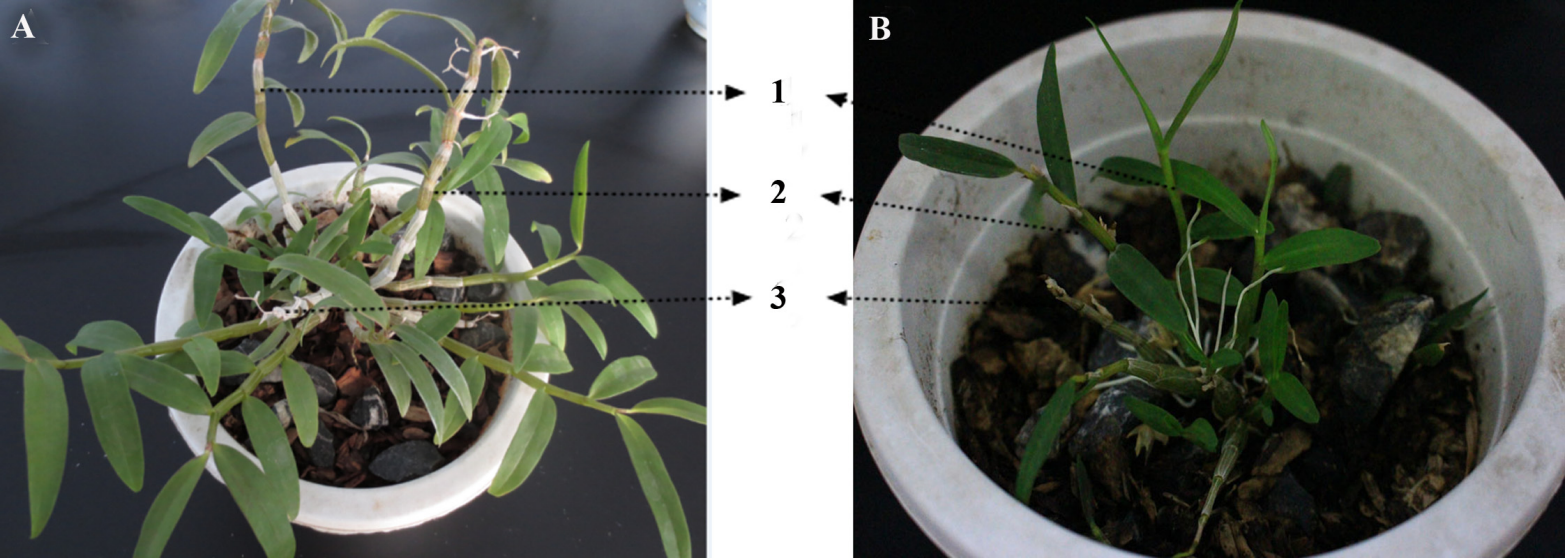
Cultivated *Dendrobium* seedlings. **(**A) Cultivated *D*. *officinale* (B) Cultivated *D*. *huoshanense*; 1, 2, and 3 represent one-,two- and three-year-old seedlings.

The experiment was conducted using one-, two- and three-year-old basin-cultured *D*. *officinale* and *D*. *huoshanense* seedlings (Fig 1) grown in a greenhouse (Hefei Anhui Mulong Mountain *Dendrobium* Biotechnology Development Co., Ltd) under the conditions of day 24°C and night 18°C, with natural light. Six replicates of each sample, including one- to three-year-old stems of the two species, were collected from the same pot. Surface soil was removed by washing with water, and the materials were dried with filter paper. Then, the samples were immediately frozen in liquid nitrogen and stored at -80°C until use for metabolomics analysis.

Methanol and chloroform (HPLC grade) were purchased from TEDIA (Fairfield, OH, USA). Pyridine was obtained from Dr. Ehrenstorfer (Augsburg, Germany). Adonitol and methoxylamine hydrochloride were purchased from Sigma-Aldrich. N,O-bis (trimethylsilyl)-trifluoroacetamide (BSTFA) containing 1% trimethylchlorosilane (TMCS) was purchased from SUPELO (Bellefonte, PA, USA). Ultra-pure water was obtained from Wahaha Group Co., Ltd. (Hangzhou, China).

### Metabolite extraction and derivatization

Metabolite extraction was performed according to the reference [12]. Each of the frozen samples (100±5 mg of fresh weight for *D*. *officinale*, 10±0.5 mg of fresh weight for *D*. *huoshanense*) was ground to a fine powder with liquid nitrogen and transferred to 10-mL centrifuge tubes. Quality control (QC) samples were used by mixing the same mass of each *Dendrobium* sample and blank samples were also prepared with empty reactions, handling with the same method as that for the real samples. Next, 1.4 mL of cold methanol (-20°C) was added to the tube and vortexed for 1 min. As an internal quantitative standard in the methanol/water phase, 60 μL of adonitol (0.2 mg/mL) was added to the tube and vortexed for 30 s. The mixture was extracted using a supersonic instrument for 30 min (40°C). Next, mixed with 750 μL of chloroform and 1.4 mL of dH_2_O (4°C) vortexed for 1 min, and centrifuged at 8000 rpm for 15 min. One milliliter of the upper phase was transferred into a fresh 1.5-mL tube and dried under a nitrogen gas stream for derivatization. First, the dried samples were dissolved in 60 μL of methoxylamine hydrochloride (20 mg/mL in pyridine), vortexed for 30 s and heated at 37°C for 120 min. Then, 60 μL of BSTFA was added, followed by heating at 25°C for 90 min. The derivatized samples were transferred into glass vials (Aglient) for GC-MS analysis.

### GC-MS analysis

The samples were analyzed using an Agilent 7890A gas chromatograph coupled to an Agilent 5975C mass spectrometer. The chromatographic separation was performed on a DB-5MS column (30 m ×250 μm × 0.25 μm). The injection volume was 1 μL, and the split ratio was 20: 1. The injector and ion source temperatures were 280°C and 250°C, respectively. The interface temperature was 250°C. The helium gas flow rate through the column was 1.0 mL/min. The temperature program began at 40°C and was held for 5 min, then increased at 10°C/min to 280°C and was maintained for 5 min. For MS detection, ions were generated by a 70-eV electron with an electron impact (EI) ionization mass spectrometric detector (MSD). Quadrupole mass spectrometry was performed using the full-scan method from 35 to 780 (m/z).

### Data analysis

Raw GC-MS data were exported into CDF format by Agilent GC/MS 6890 data analysis software and subsequently processed by XCMS (V. 1.12.1) running under the R package (V. 2.7.2). The main functions of the XCMS software include matched filtration, peak detection, peak matching and novel nonlinear retention time alignment. Internal standards and any known artefact peaks caused by column pressure, noise, solvent and derivatization procedure, were removed from the matrix. The XCMS output was further processed using Microsoft Excel 2010. The peak area of each metabolite was normalized according to the adonitol internal standard. Finally, the normalized data were imported into Simca-p software (V. 11.5) for multivariate statistical analysis, including unsupervised principal component analysis (PCA) and supervised orthogonal partial least squares discriminant analysis (OPLS-DA), and into SPSS (V. 13.0) for cluster analysis and t-tests. A statistically significant threshold of variable influence on projection values (variable influence on projection values, VIP>1) obtained from the OPLS-DA model was combined Student’s t test (t-test) (P<0.05) to identifiy discriminating metabolites [13]. Metabolites were identified by searching the commercial database NIST 11. Publicly available data from the KEGG pathway database (http://www.kegg.jp/kegg/pathway.html) were used to confirm the relationships between metabolite-metabolite correlations. Heat map analysis was performed using Multi Experiment View 4.9.

## Results

### Detection of extracts from *Dendrobium* samples

**Fig 2:**
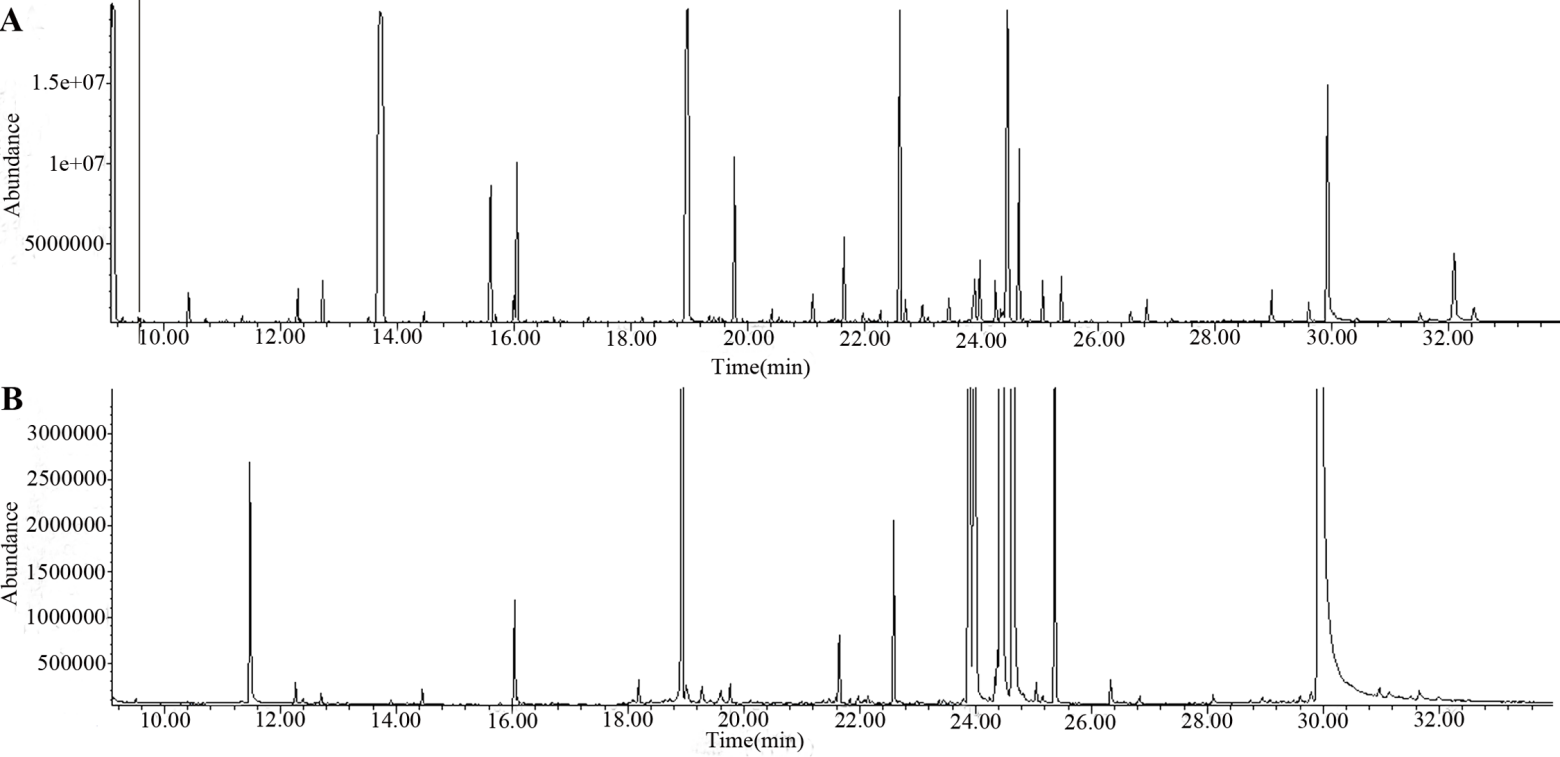
GC-MS spectra of extracts from one-year-old *D*. *officinale* and *D*. *huoshanense* stems. A and B show the spectra of methanol/water phase extracts of *D*. *officinale* and *D*. *huoshanense* one-year-old stems, respectively.

The GC-MS spectra of methanol/water phase extracts from one-year-old *D*. *officinale* and *D*. *huoshanense* stems are presented in Fig 2. In total, 544 peaks (Fig 2A) and 249 peaks (Fig 2B) were observed for *D*. *officinale* and *D*. *huoshanense*, respectively, and pretreated using AMDIS software. A total of 139 metabolites of *Dendrobium* methanol/water phase extracts were tentatively identified based on similarities of greater than 70% to mass fragments in the NIST 11 standard mass spectral databases. We classified the 139 metabolites into nine main categories: fatty acids, sugars and glycosides, organic acids, amino acids, amines and amides, alcohols, alkanes, ketones, and others (Table 1).

**Table 1:** Identification of metabolites in methanol/water phase extracts of *Dendrobium* stems.

No.	Retention time(min)	Description from best library fit	Similarity%
**Fatty acids (6 compounds)**			
1	26.68	Oleate (3TMS)	85
2	25.95	Octadecanoate (2TMS)	96
3	24.11	n-Pentadecanoate (1TMS)	90
4	22.24	Tetradecanoate (1TMS)	86
5	24.17	Hexadecanoate (1TMS)	97
6	29.33	2-Monopalmitoylglycerol (1TMS)	72
**Sugars and glycosides (26 compounds)**			
7	30.54	Trehalose (8TMS)	97
8	30.43	Mannobiose (8TMS)	87
9	29.94	Sucrose (8TMS)	96
10	29.41	Cellobiose (8TMS)	90
11	23.06	Glucose (5TMS)	95
12	22.98	Galactose (5TMS)	98
13	26.13	Mannose (5TMS)	87
14	20.50	Ribose (4TMS)	94
15	20.15	Lyxofuranose (4TMS)	90
16	22.83	Fructose (4TMS)	95
17	22.79	Sorbopyranose (5TMS)	97
18	21.48	Levoglucosan (3TMS)	89
19	20.51	Xylose (4TMS)	93
20	18.19	Erythrose (3TMS)	85
21	21.97	Arabinose (4TMS)	89
22	21.49	Sedoheptulose (6TMS)	90
23	27.68	Allose (5TMS)	89
24	30.19	Maltose (8TMS)	81
25	24.84	Tagatose (5TMS)	85
26	29.79	Turanose	87
27	24.24	Talose (5TMS)	93
28	26.19	Rhamnopyranose (4TMS)	81
29	27.45	Glucopyranoside (4TMS)	92
30	25.72	2-O-Glycerol-α-d-galactopyranoside (6TMS)	96
31	27.68	Mannopyranoside (4TMS)	86
32	25.09	Sorbopyranoside (4TMS)	98
**Organic acids (35 compounds)**			
33	19.35	2-Keto-l-gluconate (5TMS)	92
34	24.10	Gluconate (5TMS)	92
35	27.78	Glucuronate (4TMS)	91
36	24.51	Glucarate (4TMS)	91
37	24.05	Galactarate (4TMS)	87
38	11.85	Pyruvate (2TMS)	85
39	16.37	Fumarate	94
40	19.35	2-ketoglutarate (3TMS)	92
41	19.26	Threonate (3TMS)	98
42	23.37	Mannonate (4TMS)	76
43	21.52	Ribonate (4TMS)	95
44	28.46	Benzoate	87
45	19.65	Phenylacetate	95
46	15.93	Succinate (2TMS)	97
47	19.74	Tartarate (2TMS)	86
48	13.26	Oxalate	90
49	13.88	Butanoate	82
50	12.72	Propanoate	89
51	19.34	Pentanoate	85
52	13.01	Hexanoate	90
53	12.02	Acetate	87
54	17.36	Nonanoate	85
55	14.72	Carbamate	90
56	29.27	Phthalate	87
57	32.50	Isophthalate	82
58	31.30	Terephthalate	90
59	26.05	1-Naphthalenesulfonate	99
60	20.81	2-Butenedioate (2TMS)	84
61	16.54	2-Butenoate	75
62	22.67	Glutarate	86
63	22.60	1-Cyclohexene-1-carboxylic acid (3TMS)	95
64	11.89	trans-2,3-Dimethylacrylic acid	85
65	22.70	Citrate (3TMS)	92
66	27.79	Galacturonate (5TMS)	90
67	22.99	Arabino-Hexonate (4TMS)	87
**Amino acids (16 compounds)**			
68	12.75	Alanine	98
69	14.55	Valine	92
70	15.38	Norleucine	82
71	15.69	Isoleucine (1TMS)	87
72	29.19	Leucine	79
73	15.73	Proline (1TMS)	94
74	15.87	Glycine (2TMS)	94
75	16.65	Serine (2TMS)	89
76	17.01	Threonine (3TMS)	93
77	18.69	Aspartate (2TMS)	95
78	19.86	Glutamate (2TMS)	92
79	21.59	Glutamine (3TMS)	92
80	19.78	Asparagine	92
81	19.95	Phenylalanine (2TMS)	93
82	22.41	Methionine	91
83	22.55	Allylglycine	90
**Amines and amides (8 compounds)**			
84	9.66	Ethanamine	80
85	10.66	Triethylamine	87
86	20.94	1,4-Butanediamine (4TMS)	74
87	18.90	Benzamide	87
88	14.88	Benzenesulfonamide	86
89	19.97	Lysergamide	89
90	26.33	Hexadecanamide	87
91	28.11	Octadecanamide	80
**Alcohols (11 compounds)**			
92	15.44	Glycerol (3TMS)	93
93	18.62	Threitol (4TMS)	94
94	23.51	Galactitol (6TMS)	91
95	21.59	Arabitol	89
96	22.69	Myo-inositol (6TMS)	81
97	23.79	Glucitol	90
98	23.38	Mannitol (6TMS)	82
99	17.04	1,2-Propanediol	90
100	15.79	1,2-Butanediol (2TMS)	80
101	12.32	Benzenemethanol	94
102	28.12	Ethanol	88
**Alkanes (19 compounds)**			
103	10.71	Ethane	87
104	9.53	Butane	89
105	22.85	Hexane	90
106	15.36	Heptane	87
107	10.82	Octane	90
108	11.24	Decane	86
109	12.07	Nonane	87
110	12.73	Undecane	89
111	16.41	Dodecane	85
112	16.51	Tridecane	85
113	16.03	Tetradecane	84
114	19.01	Pentadecane	87
115	20.72	Hexadecane	90
116	19.06	Heptadecane	92
117	19.62	Nonadecane	77
118	18.66	Eicosane	80
119	25.79	Heneicosane	84
120	24.21	Tetracosane	70
121	21.56	Heptacosane	86
**Ketones (3 compounds)**			
122	20.76	Ethanone	98
123	23.98	Psicose (6TMS)	92
124	24.96	Propan-1-one	85
**Others (15 compounds)**			
125	24.20	Sulfurous acid	75
126	16.05	Phosphate (3:1)	95
127	15.06	Benzene	86
128	14.91	Urea	93
129	20.63	Phenol	85
130	18.44	Piperidine	73
131	10.44	α-Pinene	87
132	18.72	Cadaverine (3TMS)	70
133	28.97	Oleanitrile	90
134	14.29	1-Piperidinecarboxaldehyde	82
135	11.47	Benzonitrile	95
136	30.94	Coumarin	83
137	29.46	1,2-Benzenediol	83
138	13.32	Quinoline	87
139	29.44	Thiophene	80

### Metabolic profiling of two medicinal *Dendrobium* stems during different growth years

#### Cluster analysis of metabolites in two medicinal *Dendrobium* stems

**Fig 3:**
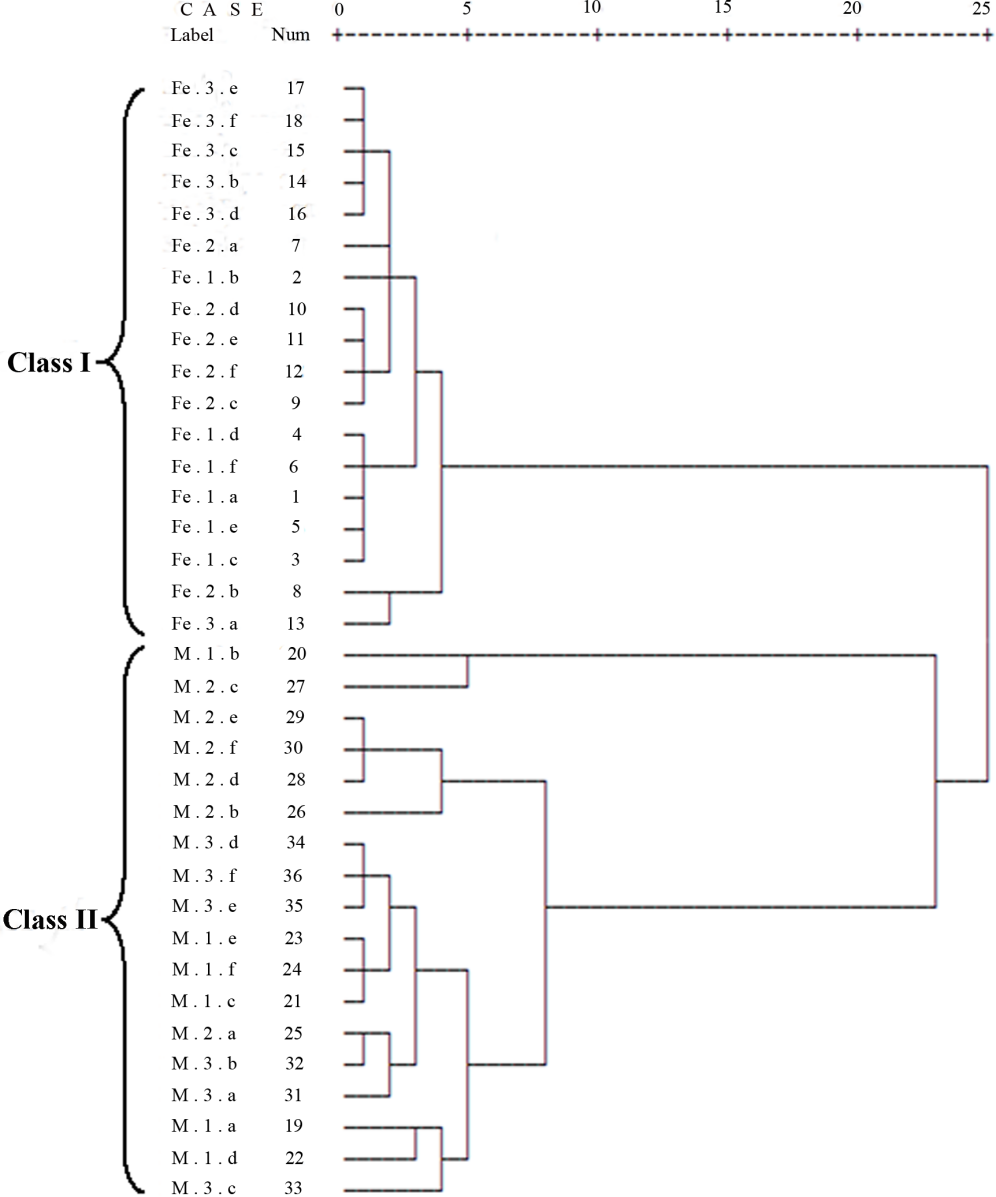
Hierarchical cluster analysis (HCA) of metabolic fingerprinters of *D*. *officinale* and *D*. *huoshanense*. “Fe” indicates *D*. *officinale*, and “M” indicates *D*. *huoshanense*. The numbers 1, 2, and 3 indicate the corresponding growth years of *Dendrobium*. The letters a, b,…, f indicate repeats of the same sample.

The metabolites in the *D*. *officinale* and *D*. *huoshanense* stems were subjected to cluster analysis using SPSS 13.0 software. *D*. *officinale* and *D*. *huoshanense* were clearly distinguished. Thirty-six *Dendrobium* samples were clustered into two major groups (Fig 3). All *D*. *officinale* samples were classified into Class I, and Class II contained 18 collections of *D*. *huoshanense*. These results demonstrate that the metabolites in cultivated *D*. *officinale* and *D*. *huoshanense* were substaintially different. The cluster analysis showed a certain influence on metabolite compositions by growth years. For example, collections of *D*. *officinale* could be generally clustered into three subgroups (one, two and three growth years), with some overlap. In order to further distinguish the two *Dendrobium* species, we need to use multivariate statistical analysis.

#### Multivariate statistical analysis of metabolites in two medicinal *Dendrobium* stems

**Fig 4:**
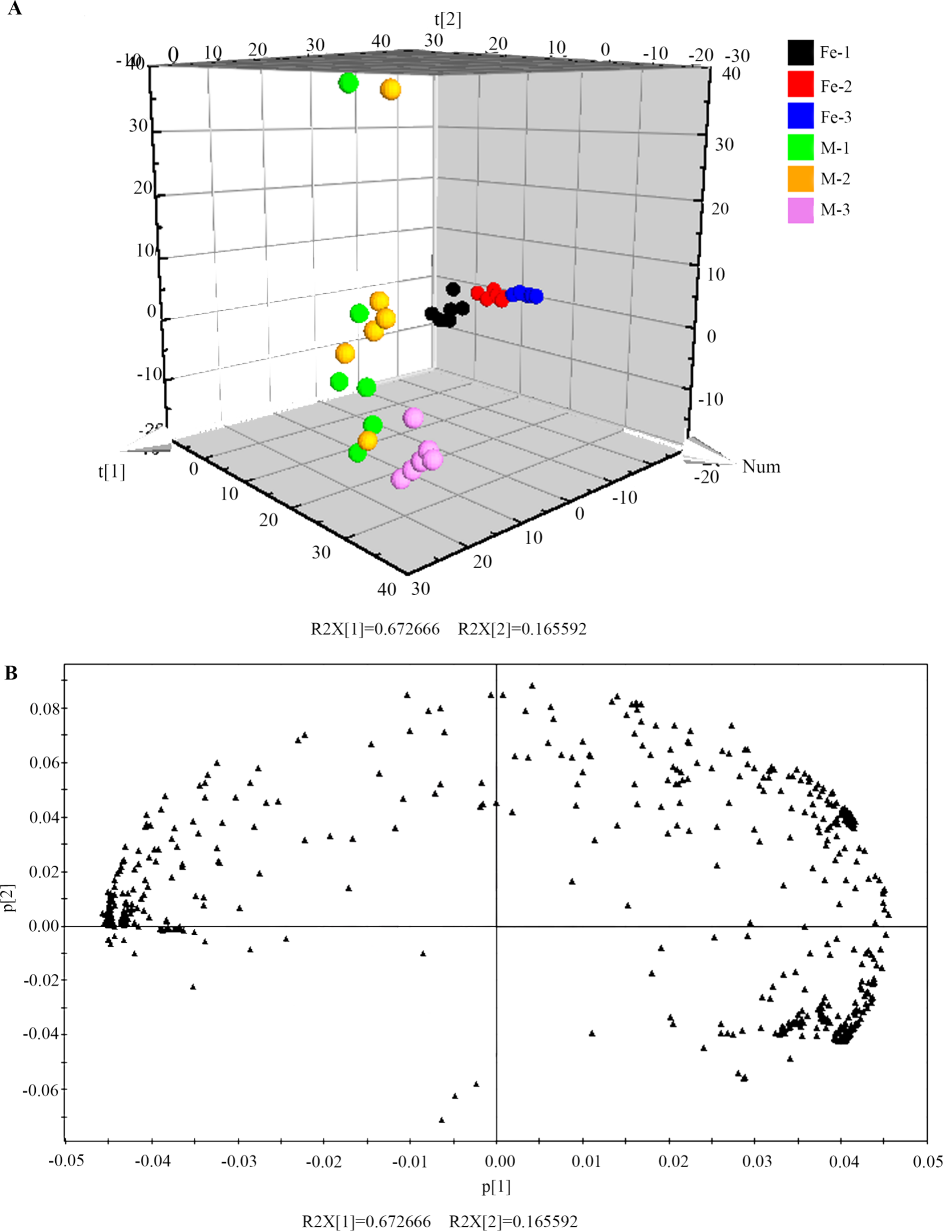
PCA scores plot and loadings plot for *D*. *officinale* and *D*. *huoshanense* with different growth years. (A) PCA scores plot for 36 *Dendrobium* collections. (B) PCA loadings plot marked by two *Dendrobium* species. “Fe” indicates *D*. *officinale*, and “M” indicates *D*. *huoshanense*. The numbers 1, 2, and 3 indicate the corresponding growth years of *Dendrobium*.

**Fig 5:**
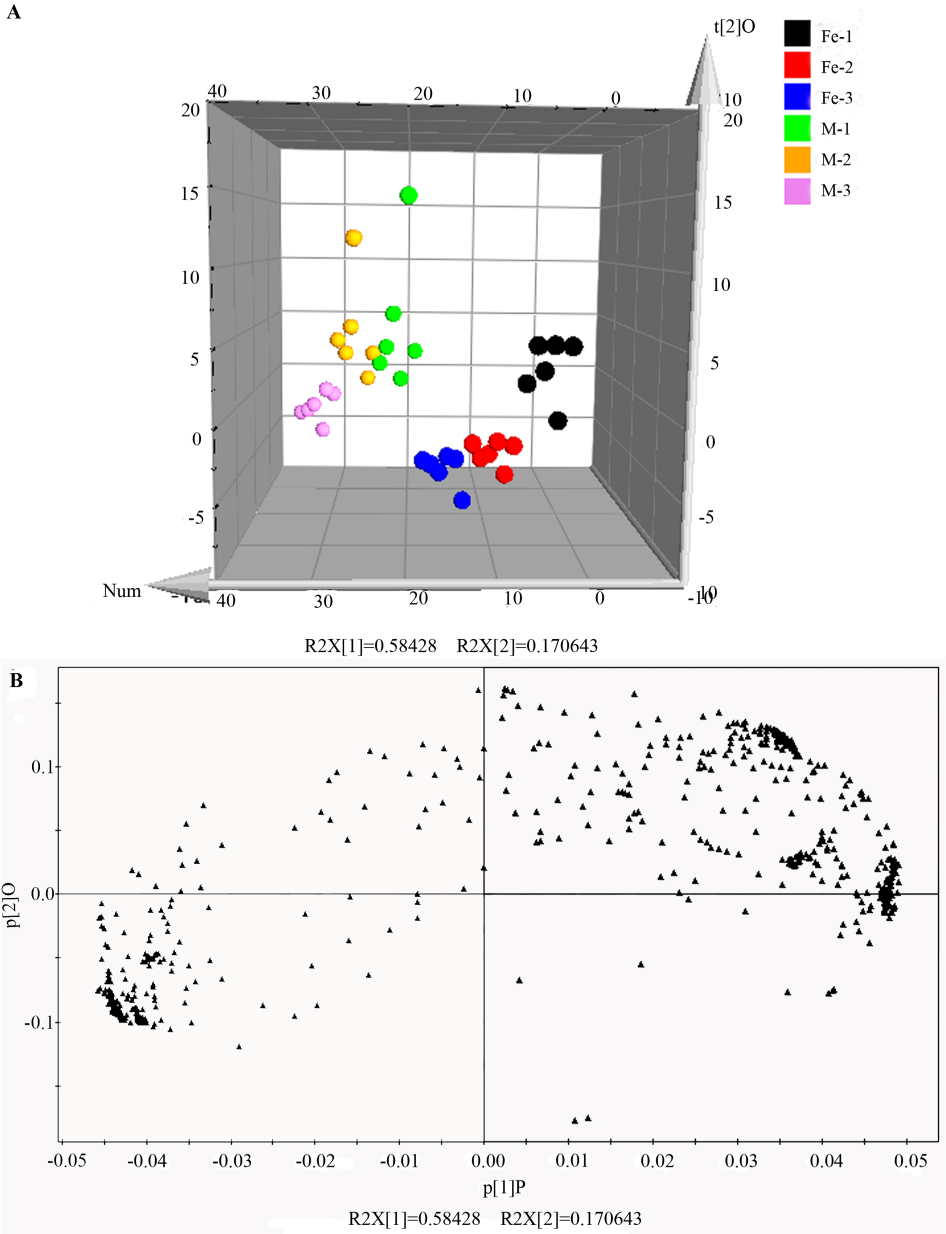
OPLS-DA scores plot and loadings plot for *D*. *officinale* and *D*. *huoshanense* with different growth years. (A) OPLS-DA scores plot for 36 *Dendrobium* collections. (B) OPLS-DA loadings plot marked by two *Dendrobium* species. “Fe” indicates *D*. *officinale*, and “M” indicates *D*. *huoshanense*. The numbers 1, 2, and 3 indicate the corresponding growth years of *Dendrobium*.

Principal component analysis (PCA) and orthogonal partial least squares discriminant analysis (OPLS-DA) of cultivated *D*. *officinale* and *D*. *huoshanense* were performed using Simca-p 11.5 software (Fig 4 and Fig 5). The PCA analysis showed a substantial difference between the two *Dendrobium* species (Fig 4), with two principal components explaining 83.8% of the total variability (67.2% and 16.6% for principal component 1 and principal component 2, respectively). Moreover, a clear separation among different growth years (one, two, and three) of *Dendrobium* samples was observed in the scores plot, and only a few of samples overlapped. In order to find the features with power to distinguish the two *Dendrobiu*m species with different growth years, OPLS-DA model (noisy information was removed prior to model building) was established with the scores plot and loadings plot shown in Fig 5. The R^2^X, R^2^Y, and Q^2^ of this model were 0.755, 0.868 and 0.861, respectively, indicating the stability and reliability of this OPLS-DA model. Obviously, in the analysis of the two *Dendrobium* species metabolites data, PCA and OPLS-DA were more powerful than cluster analysis.

**Fig 6:**
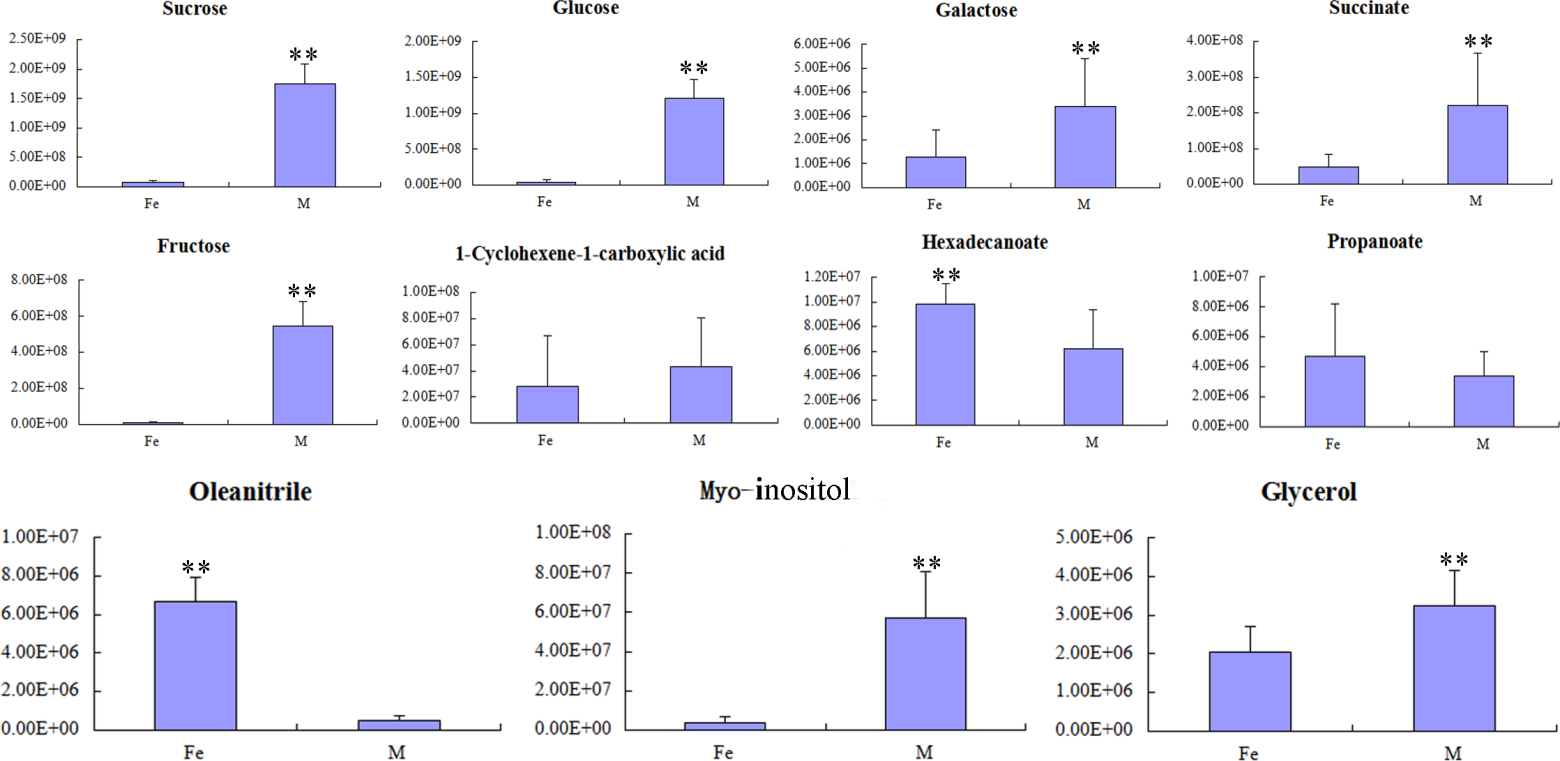
Important compounds (VIPs) that exhibited significant differences between the two *Dendrobium* species. “Fe” means *D*. *officinale*, and “M” means *D*. *huoshanense*. The bars represent metabolite peak areas across the 18 samples of each species. The error bars indicate the standard deviations of six biological repeats (including one-, two-, and three-year-old *Dendrobium* stem samples, each with six repeats). The nine candidate biomarkers were sucrose, glucose, galactose, succinate, fructose, hexadecanoate, oleanitrile, myo-inositol, and glycerol (**P<0.01, *D*. *officinale* compared to *D*. *huoshanense*).

We further employed OPLS-DA to identify the metabolites contributing significantly to the separation. Table 2 lists the top 11 metabolites (VIPs) influencing cluster formation within the methanol/water phase generated from OPLS-DA of the two *Dendrobium* species. The identified VIP components include 4 sugars (sucrose, galactose, glucose, fructose), 3 organic acids (1-cyclohexene-1-carboxylic acid, succinate, propanoate), 1 fatty acid (hexadecanoate), 2 alcohols (glycerol, myo-inositol) and 1 volatile substance (oleanitrile). The contents of sucrose, galactose, glucose, fructose, succinate, myo-inositol, and glycerol were much higher in *D*. *huoshanense* than in *D*. *officinale* (Fig 6), whereas hexadecanoate and oleanitrile levels were much lower in *D*. *huoshanense* than in *D*. *officinale*. The VIP components from OPLS-DA were combined with the t-test (P <0.05) to identify nine significantly different metabolites (sucrose, glucose, galactose, succinate, fructose, hexadecanoate, oleanitrile, myo-inositol, and glycerol) as potential biomarkers that may discriminate these two *Dendrobium* species.

**Table 2:** Metabolites identified as important variables in the projection for species discrimination.

No.	RT(min)	Name	VIP	p-value	FDR-value
1	29.94	Sucrose	1.192	1.07E-15	6.30E-15
2	24.49	Glucose	1.191	3.27E-27	3.27E-25
3	24.31	Galactose	1.178	1.59E-15	1.47E-18
4	18.95	Succinate	1.163	1.08E-16	7.35E-16
5	24.08	Fructose	1.161	2.98E-22	8.70E-21
6	22.61	1-Cyclohexene-1-carboxylic acid	1.148	0.06	0.06
7	25.04	Hexadecanoate	1.145	7.98E-08	1.09E-07
8	12.73	Propanoate	1.145	0.06	0.06
9	28.99	Oleanitrile	1.140	2.35E-28	8.24E-26
10	25.37	Myo-inositol	1.118	8.2E-10	1.24E- 09
11	16.05	Glycerol	1.099	1.79E-15	9.43E-15

RT, retention time; VIP, variable importance in the projection; p-value and FDR-value indicate the significance and false discovery rate of difference of the relative metabolite levels between *D*. *officinale* and *D*. *huoshanense*, respectively.

#### Metabolites levels in the stems of two medicinal *Dendrobium* species during different growth years

**Fig 7:**
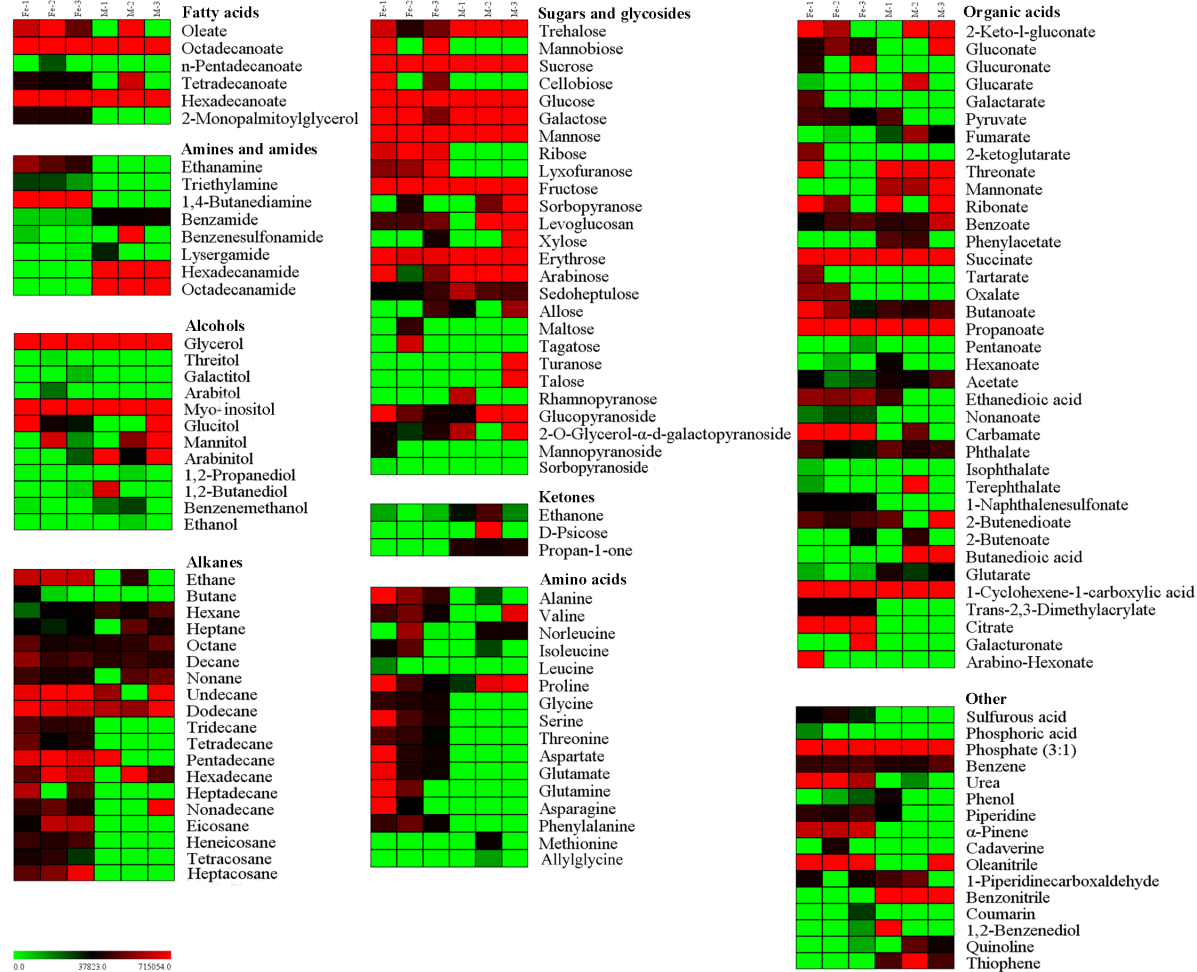
Heat map of major metabolites during different growth stages of *D*. *officinale* and *D*. *huoshanense*. All data are provided in S1 Table. The metabolite peak areas in each sample represent the average peak areas. The lowest figures are in green, and the highest figures are in red. “Fe” and “M” refer to *D*. *officinale* and *D*. *huoshanense*, respectively. The numbers 1, 2, and 3 represent one, two, and three growth years.

Raw GC-MS data were pretreated, and the peak area of each metabolite was obtained. The software Multi Experiment View 4.9 was used to construct a heat map, displaying the changes in metabolite content between *D*. *officinale* and *D*. *huoshanense* in one-, two-, and three-year-old stems (Fig 7). The result suggested that the levels of the majority of amino acids were higher in *D*. *officinale* than in *D*. *huoshanense*. Amino acids decreased in *D*. *officinale* during growth (from one to three years). By contrast, amino acids were maintained at low levels during three years of growth in *D*. *huoshanense*, with only valine and proline exhibiting higher levels in three-year-old stems. The profiles of changes in sugar and glycoside levels were obvious in both *D*. *officinale* and *D*. *huoshanense*. The total amount of sugars was higher in *D*. *huoshanense* than *D*. *officinale*. Sucrose, glucose, mannose, fructose and erythrose maintained constant high levels during the three-year growth period in both *Dendrobium* species, whereas galactose and trehalose reached their highest levels in *D*. *officinale* during the first growth year and then decreased in the next two years. For *D*. *huoshanense*, sugar and glycoside levels either remained constantly high or increased from one to three growth years. The organic acids propanoate, succinate, and 1-cyclohexene-1-carboxylic acid all remained at a high level during the three growth years. In *D*. *officinale*, 2-keto-l-gluconate, 2-ketoglutarate, glutarate, ribonate and arabino-hexonate decreased throughout the three-year growth period. In *D*. *huoshanense*, 2-keto-l-gluconate, gluconate, acetate, 2-butenoate and benzoate increased significantly during the three years. Fatty acids and ketones did not vary during the growth stage. Alcohols other than glycerol and myo-inositol remained at high levels in both *Dendrobium* species. Other alcohols exhibited different changes between *D*. *officinale* and *D*. *huoshanense*. For example, arabitol and 1,2-butanediol increased in *D*. *officinale* but decreased in *D*. *huoshanense*.

### Construction of metabolic profiling between two medicinal *Dendrobium* species

**Fig 8:**
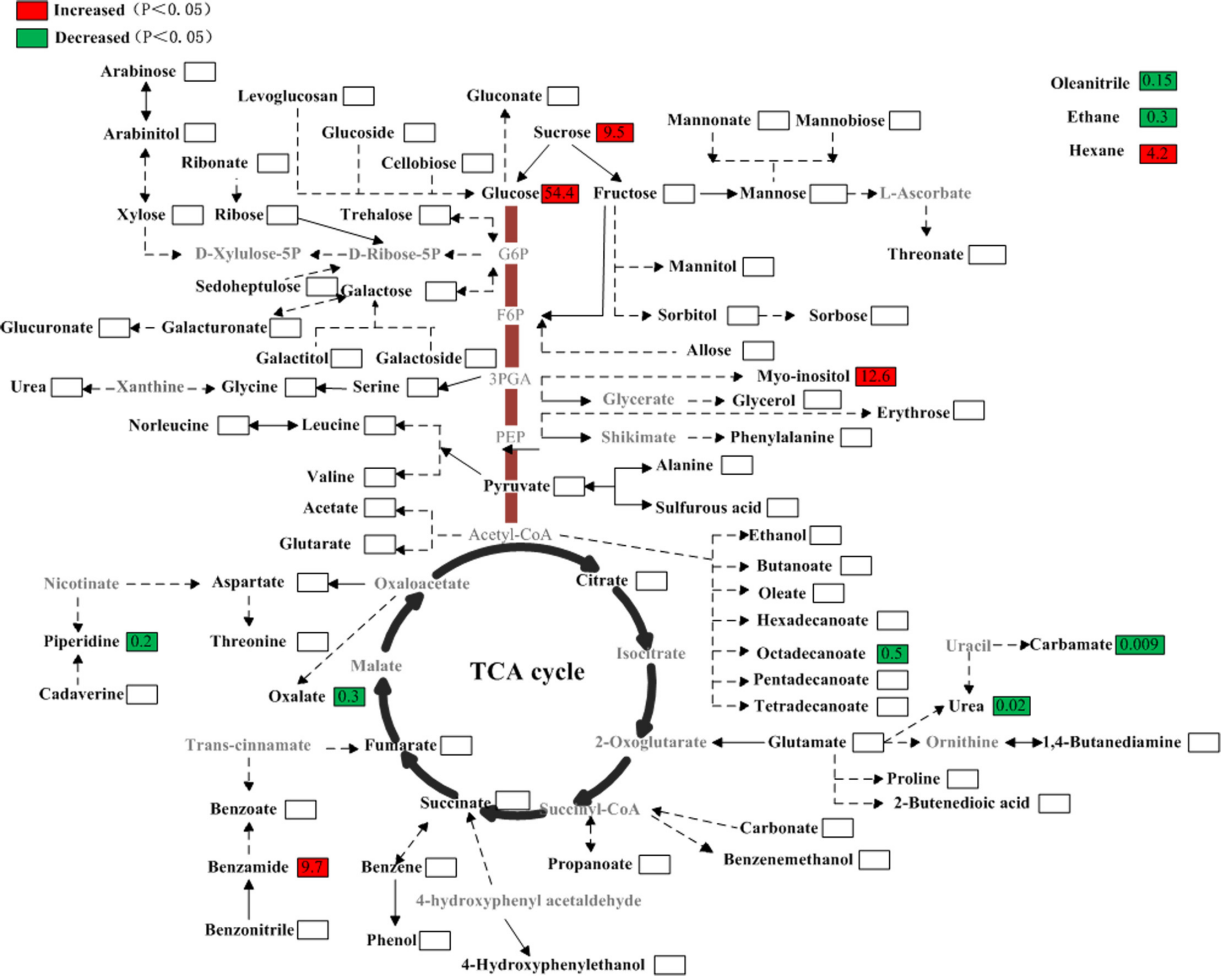
Comparisions of metabolite levels in *D*. *officinale* and *D*. *huoshanense*. The ratios in the red or green bar indicate high or low relative metabolite peak areas, respectively, of cultivated *D*. *huoshanense* compared to *D*. *officinale*. The level of significance was set at P<0.05. The metabolites in gray characters were undetectable. G6P, glucose-6-phosphate; F6P, fructose-6-phosphate; 3PGA, 3-phosphoglycerate; PEP, phosphoenolpyruvic acid.

The functions of the identified metabolites in the main plant metabolic pathways network were examined (Fig 8). As compared with those in *D*. *officinale*, the contents of sucrose, glucose, myo-inositol, hexane and benzamide in *D*. *huoshanense* increased 9.5-fold, 54.4-fold, 12.6-fold, 4.2-fold and 9.7-fold, respectively. Whereas, the contents of piperidine, oxalate, octadecanoate, urea, carbamate, ethane and oleanitrile decreased 0.2-fold, 0.3-fold, 0.5-fold, 0.02-fold, 0.009-fold, 0.3-fold and 0.15-fold, correspondingly. As shown above, most soluble sugars showed significant increases in *D*. *huoshanense*, which may suggest much higher freezing tolerance in *D*. *huoshanense* [14]. In *D*. *officinale*, the content of piperidine was much higher than that in *D*. *huoshanense*.

## Discussion

Metabolomics has been applied extensively to plants [15–17], such as the identification of metabolite changes, the identification of differences in metabolites between wild type and mutant plants [18–21]. The use of metabolomics to study *Dendrobium* metabolites has not been reported. We preliminarily constructed a metabolomics platform and analyzed the metabolites in the methanol/water phase of cultivated *D*. *officinale* and *D*. *huoshanense* stems collected during different growth years.

### Analysis of *Dendrobium* metabolic profiling provides an important basis for species identification

Cluster analysis of the methanol/water phase metabolites of *Dendrobium* distinguished the two *Dendrobium* species. *D*. *officinale* and *D*. *huoshanense* were classified into two clusters, Class I and Class II, respectively. PCA and OPLS-DA, two multivariate statistical analysis methods widely used in metabolomics, both not only clearly separated *D*. *officinale* and *D*. *huoshanense* but also distinguished different growth years of each *Dendrobium* species. VIP components combined with the t-test (P<0.05), in which significantly different metabolites were selected as potential biomarkers (sucrose, glucose, galactose, succinate, fructose, hexadecanoate, oleanitrile, myo-inositol, and glycerol), provide an important method for *Dendrobium* identification. Consistent with our results, Yuan H reported that the relative peak area of glucose could be used as a foundation for *Dendrobium* identification using a pre-column derivatization HPLC method [22]. Currently, oleanitrile has not been reported in *Dendrobium* species. In our study, oleanitrile was one of the biomarkers of *Dendrobium*, which suggests the metabolomics technology platform we constructed is relatively comprehensive.

### Analysis of *Dendrobium* metabolic profiling provides an important basis for quality control of medicinal *Dendrobium*

The main chemical components in medicinal *Dendrobium* are polysaccharides and alkaliods, with multiple biological activities [23]. Polysaccharide content has been used to determine the medicinal quality of *Dendrobium* [24]. The heat map and 11 VIP components confirmed the higher sugar content in *D*. *huoshanense* than in *D*. *officinale* (Fig 7 and Table 2), which indicates that *D*. *huoshanense* exhibits better quality. According to the metabolic profiling of *Dendrobium*, we suppose that *D*. *officinale* may have piperidine alkaliod because of its high piperidine content [25]. However, piperidine alkaliod has not been successfully annotated. It’s probable that we just detected the metabolites in polar phase of *Dendrobium*. The metabolites from *Dendrobium* non-polar phase should also be detected since most alkaloids are fat-souble in plants. Besides, most of the secondary metabolites are thermally labile and unsuitable for GC-MS analysis.

During the three growth years, the total sugar content first decreased and then increased. These changes may reflect the basic physical transformation and energy metabolism in the *Dendrobium* vegetative phase. Amino acids levels decreased over the growth years because basic amino acids are nitrogen-containing precursors that are involved in the biosynthesis of a variety of secondary metabolites in plants. Based on the accumulated metabolites in *Dendrobium*, the optimal harvest time is in the third year [26]. Cluster analysis and multivariate statistical analysis of *Dendrobium* metabolites discriminated different *Dendrobium* species and provided a new basis for identification and quality control.

In our study, the metabolites in *Dendrobium* stems were analyzed using GC-MS, and the majority of the metabolites examined were primary metabolites. In future work, we will combine metabolomics, transcriptomics and proteomics technologies to further study secondary metabolites, particularly pharmaceutically effective ingredients in *Dendrobium* and synthesis mechanisms.

## Supporting Information

S1 Table Major metabolites of *D*. *officinale* and *D*. *huoshanense* during different growth years. (XLS)
